# Faux anévrysme de l'artère humérale suite à une plaie par arme à feux

**DOI:** 10.11604/pamj.2015.22.212.7798

**Published:** 2015-11-09

**Authors:** Melek Ben Mrad, Nizar Elleuch

**Affiliations:** 1 Service de Chirurgie Cardio-vasculaire, Hopital La Rabta, Faculté de Médecine de Tunis, Université Tunis EL Manar, Tunisie

**Keywords:** Faux anévrysme, artère humérale, arme à feu, pseudo-aneurysm, brachial artery, firearm

## Image en medicine

Les plaies par balles peuvent toucher les trajets artériels, elles sont responsables souvent d'un double syndrome: hémorragique et ischémique. Dans ces cas les patients sont généralement opérés en urgence vu l'urgence vitale ou/et fonctionnelle. Parfois les lésions artérielles passent inaperçus. C'est le cas des lésions latérales et partielles, elles évoluent alors vers la formation d'anévrysmes artériels. Nous rapportons le cas d'un jeune âgé de 20 ans, victime lors d'un combat armé d'une plaie par balle avec un point d'entrée au niveau du thorax. Le patient est sorti indemne initialement mais il a développé dans les jours qui ont suivi une masse au niveau de la face interne du bras gauche. Devant l'apparition d'une gêne neurologique à type de paresthésies au niveau de la main gauche, le patient nous a consultés. Le diagnostic de faux anévrysme huméral avec compression nerveuse a été retenu sur les données cliniques et scannographiques (A). Le patient a été opéré, avec contrôle premier de l'anévrysme (B), résection de l'artère humérale atteinte et du tissu anévrysmal (C), et rétablissement de la continuité artérielle par un pontage veineux en utilisant la veine basilique prélevé au même site opératoire (D). L'évolution initiale été bonne avec disparition de la gêne neurologique. Le patient se porte bien deux ans après l'intervention. Le diagnostic du faux anévrysme artériel doit être évoqué devant toute masse périphérique sur un trajet artériel même lorsque le point d'entrée est loin du site.

**Figure 1:**
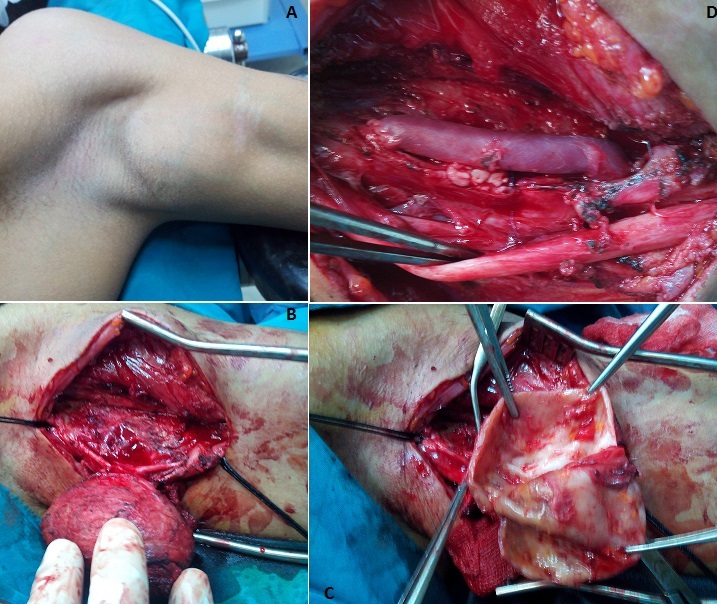
Images peropératoires: A) masse de la face interne de la partie supérieure du bras gauche; B) dissection du faux anévrysme; C) ouverture du faux anévrysme avec résection du tissu anévrysmal; D) pontage huméro-huméral par veine basilique homolatérale

